# Generation of hypoxanthine phosphoribosyltransferase gene knockout rabbits by homologous recombination and gene trapping through somatic cell nuclear transfer

**DOI:** 10.1038/srep16023

**Published:** 2015-11-02

**Authors:** Mingru Yin, Weihua Jiang, Zhenfu Fang, Pengcheng Kong, Fengying Xing, Yao Li, Xuejin Chen, Shangang Li

**Affiliations:** 1 Department of Laboratory Animal Science, School of Medicine, Shanghai Jiao Tong University, 200025 Shanghai, China

## Abstract

The rabbit is a common animal model that has been employed in studies on various human disorders, and the generation of genetically modified rabbit lines is highly desirable. Female rabbits have been successfully cloned from cumulus cells, and the somatic cell nuclear transfer (SCNT) technology is well established. The present study generated hypoxanthine phosphoribosyltransferase (*HPRT*) gene knockout rabbits using recombinant adeno-associated virus-mediated homologous recombination and SCNT. Gene trap strategies were employed to enhance the gene targeting rates. The male and female gene knockout fibroblast cell lines were derived by different strategies. When male *HPRT* knockout cells were used for SCNT, no live rabbits were obtained. However, when female *HPRT*^+/−^ cells were used for SCNT, live, healthy rabbits were generated. The cloned *HPRT*^+/−^ rabbits were fertile at maturity. We demonstrate a new technique to produce gene-targeted rabbits. This approach may also be used in the genetic manipulation of different genes or in other species.

Animals with specific genetic mutations are powerful tools for investigating gene function and human diseases. Various genetic alterations have been induced in mice to improve our understanding of both human disease pathogenesis and the development of new treatment regimens. However, mice fail to replicate the phenotype of several diseases, including Lesch-Nyhan syndrome^1^,^2^ and cystic fibrosis^3^. Additional genetically manipulated animal species, including nonhuman primates, pigs, ferrets, hamsters, and rabbits, are thus warranted for specific disorders. Traditional gene recombination is based on embryonic stem cell (ESC) technology. However, despite several attempts in various species, mammalian germ line-transferred ESCs have only been developed in the mouse^4^,^5^ and the rat^6^. Somatic cell nuclear transfer (SCNT) technology, which was first employed to clone sheep in 1997^7^, is another demonstrated method of generating an adult from a single cell, regardless of whether it is a transgenic^8^,^9^ or gene-targeted model^10^. SCNT has succeeded in creating genetically manipulated animals in various species, including sheep^10^, pigs^11^,^12^ and ferrets^13^. In recent years, several new techniques in producing genetically mutated mice, rats, monkeys, rabbits, or cell lines have been established, including zinc finger nucleases (ZFN), transcription factor activator-like effector nucleases (TALENs), and CRISPR/Cas9 gene editing systems^14^,^15^,^16^,^17^,^18^,^19^. Although each genetic manipulation technique has different benefits and disadvantages^20^,^21^, establishing gene knockout animals via homologous recombination is still desirable because it could facilitate both the expression of an exogenous protein using an endogenous promoter and the development of a strategy for generating conditional gene knockouts.

Rabbits are phylogenetically closer to primates than they are to rodents, and they are physically large enough to permit the non-lethal monitoring of the physiological changes. Rabbits are also the traditional animal models that are employed for the study of lipoproteins and atherosclerosis because their lipid metabolism is similar to that of humans. Furthermore, a number of spontaneous and transgenic rabbit models have been studied^19^. Given their extensive use in research, the creation of genetically modified rabbit lines is highly desirable. Female rabbits have been successfully cloned from freshly isolated cumulus cells^22^, and the SCNT technology in rabbits has been the focus of much research^23^. Transgenic cloned rabbits have been produced from fibroblasts and mesenchymal stem cells^24^,^25^. However, somatic cell proliferation is limited, and low levels of homologous gene recombination occur in these cells. Therefore, isolating the genetically manipulated cells may impede the production of gene-modified rabbits by SCNT, and no gene-modified rabbit cell lines have been reported to date.

Hypoxanthine phosphoribosyltransferase (HPRT) is an enzyme that is essential for the metabolic salvage of purines in mammalian cells. A deficiency in HPRT causes the clinical disorders of Lesch-Nyhan disease (LND) and gouty arthritis in human males^26^,^27^. An *HPRT* gene knockout animal model that could mimic Lesch-Nyhan syndrome is desired; however, to date, no ideal model has been reported. The *HPRT* gene is located on the X chromosome and cells deficient in the enzyme are capable of avoiding the cytotoxic effects of 6-thioguanine (6-TG) exposure. Thus, the *HPRT* gene has been a useful selective target^28^,^29^.

In the present study, we generated *HPRT* gene knockout rabbits by recombinant adeno-associated virus (rAAV)-mediated homologous recombination (HR) and gene trapping. AAV is a 4.7 kb single-stranded DNA virus that was developed as a transducing vector capable of integration into mammalian chromosomes^30^. rAAV vectors were first successfully used in site-specific genetic modification^29^ to target transgene insertion into the chromosomes of cultured human cells^28^. This rAAV vector delivery system was well established for CFTR knockout in pigs^12^ and ferrets^13^. Gene trapping is a traditional, efficient system for simultaneously characterizing gene function, sequence and expression^31^. Various strategies for gene trapping have been extensively performed on mouse ES cells. Gene trap strategies were used in the present study to enhance the gene targeting rates. *HPRT* gene knockout fibroblast cell lines of both genders were derived using different strategies and employed in SCNT, and healthy *HPRT* gene knockout rabbits were obtained from the female lines. This approach may also be used to facilitate the efficient manipulation of different genes or in other species.

## Results

### rAAV vector infects rabbit fibroblasts

To test whether rAAV vectors could be introduced into rabbit fibroblasts, rAAV-hrGFP, pAAV-RC, and pHELPER were mixed and introduced into 293A cells using the calcium phosphate cell transfection method. Three days after transfection, the rAAV-hrGFP vector was packaged into 293A cells, and GFP fluorescence was identified in most cells (Supplementary Fig. S1). The rAAV-hrGFP viral particles were extracted and delivered into a male rabbit fibroblast cell line using the same method as that used in the gene targeting vectors. Seven days after transfection, GFP fluorescence was identified in approximately 40% of fibroblasts (Supplementary Fig. S1), which indicated that rabbit fibroblasts are susceptible to rAAV infection and that our targeting protocol was effective.

### Introducing the HPRT gene knockout construct into fibroblasts using rAAV vectors

**Figure 1:**
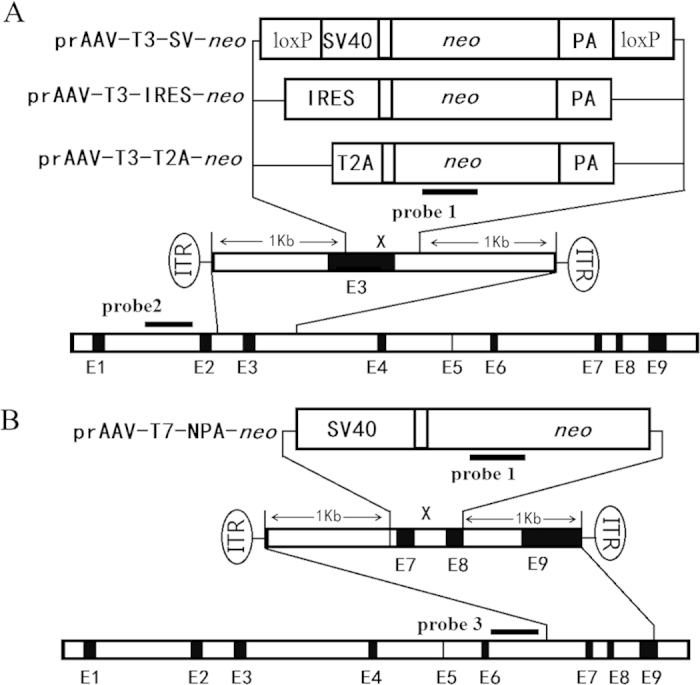
Structure of the wild-type *HPRT* locus and design of the rAAV-targeting vectors. (**A**) Vector targeting of the 3′ part of exon 3 and intron 3; the different *Neo* constructs are shown in the diagram (for details, see the Materials and Methods) and are identified as T3SV, T3IR and T3T2A, respectively. (**B**) Vectors targeting exons 7 and 8 locus; the *Neo* without polyA constructs are depicted (for details, see the Materials and Methods) and designated as T7NPA. The probes used in Southern analysis are shown.

Four variants of neomycin phosphotransferase (*Neo*) cassettes derived from distinct plasmids were used as vectors: SV-neo, IRES-neo, T2A-neo (Fig. 1A), and NPA-neo (Fig. 1B). SV-neo is a fragment that includes the SV40 promoter and *Neo*. IRES-neo is a traditional vector used in promoter trapping, containing an internal ribosome entry site (IRES), which is a nucleotide sequence that facilitates translation initiation at the middle of a messenger RNA (mRNA) sequence. To increase the efficiency of rAAV delivery by shortening the length of the vector, a T2A element (coding for 18 amino acids) was employed in the T2A-neo vector. T2A is one of the 2A self-cleaving peptides^32^ and is widely utilized in gene co-expression studies^33^. To broaden the gene trap strategy to the genes that are not expressed in somatic cells, we designed a polyadenylation (polyA)-trap vector named NPA-neo, in which the SV40 promoter drives the expression of *Neo* that lacks a polyA signal, as previously reported^34^. SV-neo, IRES-neo, and T2A-neo underwent replacement of the 3′ part of exon 3 and the 5′ part of intron 3. Three plasmids were produced: prAAV-T3-SV-neo (T3SV), prAAV-T3-IRES-neo (T3IR), and prAAV-T3-T2A-neo (T3T2A). NPA-neo replaced the sequence comprising exons 7 and 8, and one plasmid was constructed and designated as prAAV-T7-NPA-neo (T7NPA). Each of the rAAV targeting constructs was packaged.

**Figure 2:**
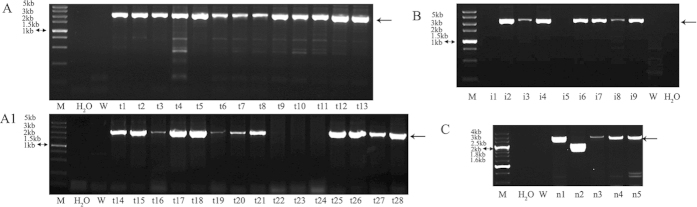
PCR detection of the male *HPRT*-null cell lines using primers over the HR arm. (**A**,**A1**) PCR analysis of the DNA isolated from male 6-TG-resistant rabbit fibroblasts stably transfected with T3T2A; the arrows indicate the target fragments of approximately 2.5 kb. t22, t23 and t24 were the negative cell lines. (**B**) PCR analysis of the DNA isolated from male 6-TG-resistant rabbit fibroblasts stably transfected with T3IR; the arrow indicates the target fragments of approximately 2.5 kb. i1, i5 were the negative cell lines. (**C**) PCR analysis of the DNA isolated from male 6-TG-resistant rabbit fibroblasts stably transfected with T7NPA; the arrow indicates the target fragments of approximately 3 kb. n2 was the negative cell line. W, wild-type rabbit fibroblasts (negative control). The primers over the HR arm were listed in Table S1

**Figure 3:**
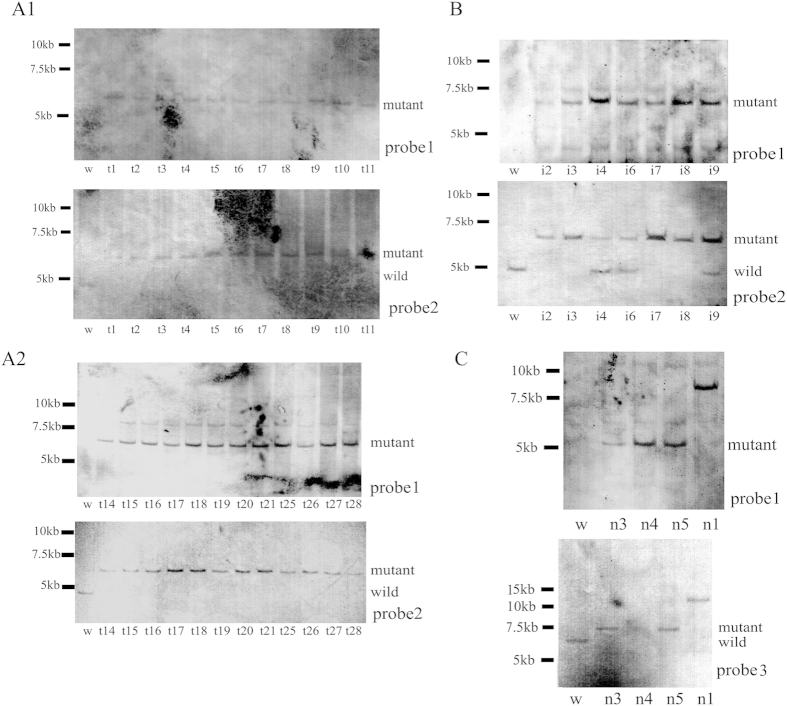
Southern blotting analysis of the PCR-positive cell clones derived from the male *HPRT*-null cell lines using different probes. (**A1**,**A2**) gDNA from 23 cell lines transfected with T3T2A and digested with *Hind*III; the labelled mutant fragment is 5997 bp, and the labelled wild-type fragment is 4,579 bp. (**B**) gDNA from 7 cell lines transfected with T3IR and digested with *EcoT22*I; the labelled mutant fragment is 6,839 bp, and the labelled wild-type fragment is 4,870 bp. Some cell lines (i4, i6, and i9) were complex clones that contained wild-type and mutant cells. (**C**) gDNA from 4 cell lines transfected with T7NPA and digested with *Hpa*I (probe 1); the labelled mutant fragment is 5,546 bp (probe 1). gDNA from 4 cell lines transfected with T7NPA and digested with *EcoRV and EcoRI* (probe 3); the labelled mutant fragment is 7,590 bp (probe 3), and the labelled wild-type fragment is 6,620 bp (probe 3). W, wild-type gDNA (negative control); Probe 1 for *Neo*; Probe 2 and probe 3 for *HPRT*.

Male fibroblasts were used because the *HPRT* locus is X-chromosome linked (i.e., hemizygous in males), and no HPRT was produced when the single-copy *HPRT* gene was knocked out. The G418-positive male cells could also be selected by 6-TG exposure. When the four different vectors were each introduced into male fibroblasts, several G418-resistant clones were generated. After 6-TG selection and PCR analysis, three vector-derived *HPRT* knockout clones were identified: T3T2A [25 targeted clones, 2 clones (t12, t13) were lost during the expansion; Fig. 2A,A1], T3IR (7 targeted clones; Fig. 2B), and T7NPA (4 targeted clones, Fig. 2C). No *HPRT* knockout cell lines were derived from the T3SV vector (Table 1). The *HPRT* knockout clones were also confirmed by Southern blotting analysis using Probes 1, 2 and 3 (Fig. 3). However, one cell clone (n1) from the T7NPA vector had a larger than expected band after restriction endonuclease digestion and could survive under 6-TG when hybridized with Probe 3, and 3 clones from T3IR were mixed clones that showed two bands when hybridized with Probe 2. Several *HPRT* knockout cell lines were selected for protein expression analysis by western blotting (Supplementary Fig. S2), and the results confirmed the findings of Southern analysis.

**Figure 4:**
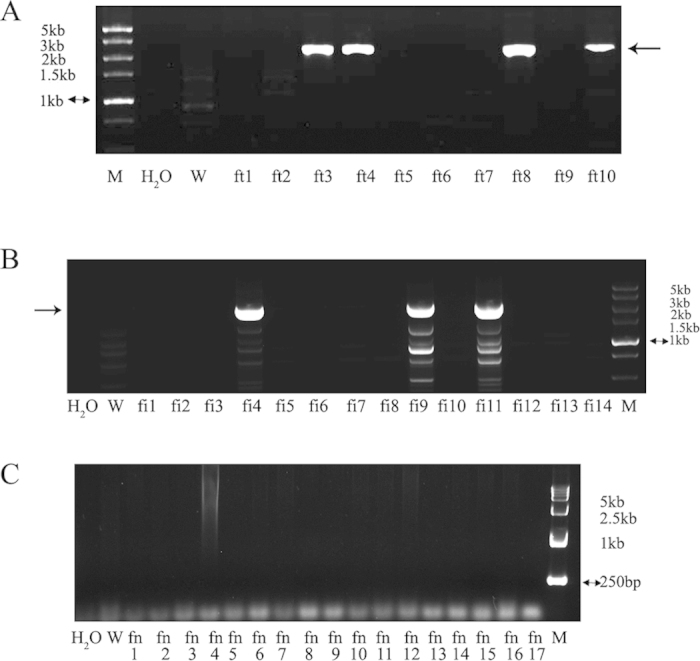
PCR detection of the female *HPRT*^+/−^ cell lines using primers over the HR arm. (**A**) PCR analysis of the gDNA isolated from female G418-resistant rabbit fibroblasts stably transfected with T3T2A; the arrow indicates the target fragments of approximately 2.5 kb. ft3, ft4, ft8, and ft10 are the positive cell lines. (**B**) PCR analysis of the gDNA isolated from female G418-resistant rabbit fibroblasts stably transfected with T3IR; the arrow indicates the target fragments of approximately 2.5 kb. fi4, fi9, and fi11 are the positive cell lines. (**C**) PCR analysis of the gDNA isolated from female G418-resistant rabbit fibroblasts stably transfected with T7NPA; no positive cell lines were obtained. W, wild-type rabbit fibroblasts (negative control).

**Figure 5:**
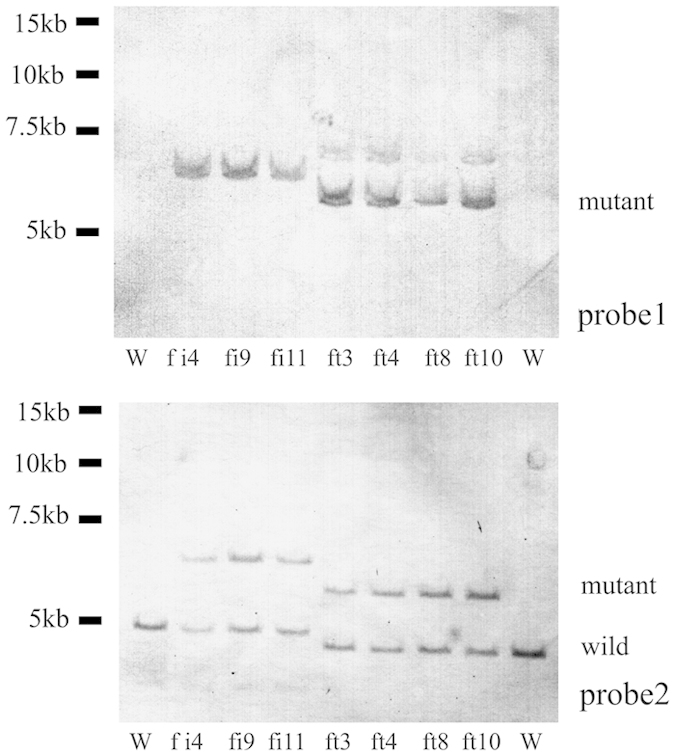
Southern blotting analysis of the PCR-positive cell clones derived from the female *HPRT*^+/−^ cell lines using different probes. The fi cell lines transfected with T3IR and digested with *EcoT22*I; the labelled mutant fragment is 6,839 bp, and the labelled wild-type fragment is 4,870 bp. The ft cell lines transfected with T3T2A and digested with *Hind*III; the labelled mutant fragment is 5,997 bp, and the labelled wild-type fragment is 4,579 bp. W, wild-type gDNA (negative control); Probe 1 for *Neo* and Probe 2 for *HPRT*.

Poor results were obtained when gene knockout male fibroblasts were used in SCNT; thus, female fibroblasts were used for the *HPRT* knockout experiments. When the three vectors, T3IR, T3T2A, and T7NPA, were transfected into a female fibroblast line, the number of G418-resistant clones obtained was low (Table 1). By PCR analysis, 4 and 3 *HPRT* knockout clones were derived from the T3T2A and T3IR vectors (Fig. 4A,B), respectively (Table 1). No *HPRT* knockout clones were isolated from the T7NPA vector (Fig. 4C). The results of PCR (Fig. 4A,B) and Southern blotting (Fig. 5) analyses agreed with this finding.

### Generation of HPRT gene knockout rabbits by SCNT

**Figure 6:**
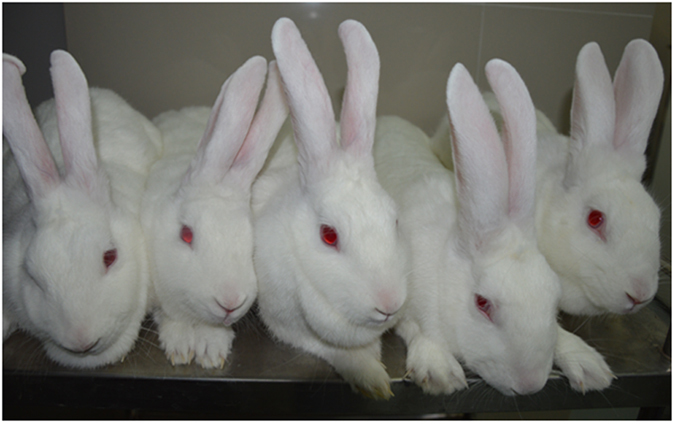
Five cloned *HPRT*^+/−^ rabbits.

To produce *HPRT* knockout rabbits, male *HPRT*-null cell lines harbouring T2A-neo were used as nuclear donors for SCNT. However, as shown in Table 2, when 276 cloned embryos were transferred into 11 recipients, only four stillborn foetuses that were retarded at gestation day 20 were delivered; no live kittens were born. Because the impact of HPRT deficiency on the success of nuclear transfer cannot be excluded, we used heterozygous *HPRT*^+/−^ female fibroblasts for the subsequent SCNT experiments. When 784 cloned embryos were transferred into 38 recipients, 24 full-term developed foetuses were delivered, of which 18 survived post-partum. Thirteen of the cloned *HPRT*^+/−^ rabbits died 3 days after birth due to dysontogenesis or unknown reasons. Five cloned *HPRT*^+/−^ rabbits from three recipients (1, 1, and 3) survived (Table 2 and Fig. 6).

### Isolation, culture, and gene analysis of fibroblasts from HPRT^+/−^ rabbits

**Figure 7:**
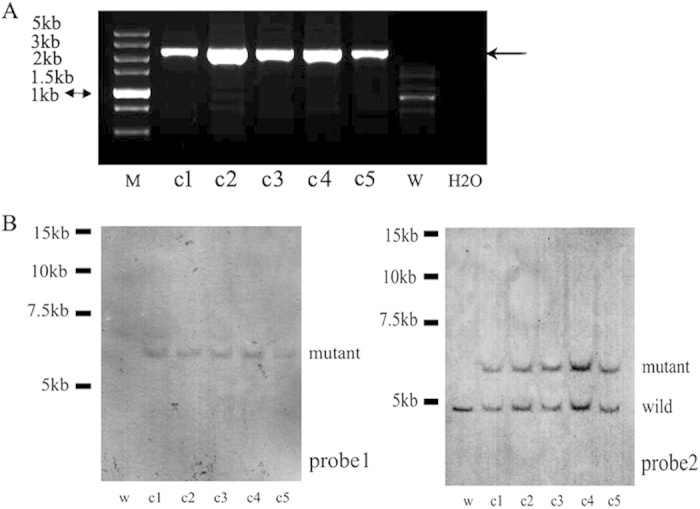
PCR analysis of the 5 cloned *HPRT*^+/−^ rabbits using primers over the HR arm, and Southern blotting analysis of the gDNA from the cloned *HPRT*^+/−^ rabbits using different probes. (**A**) PCR analysis of the 5 cloned *HPRT*^+/−^ rabbits using primers over the HR arm; the arrow indicates the target fragments of approximately 2.5 kb; W, wild-type rabbit fibroblasts (negative control). (**B**) Southern blotting analysis of the *Hind*III-digested gDNA from the five cloned *HPRT*^+/−^ rabbits; W, wild-type gDNA (negative control). The labelled mutant fragment is 5,997 bp; the labelled wild-type fragment is 4,579 bp. Probe 1 for *Neo* and Probe 2 for *HPRT*.

Fibroblasts were isolated from five 3- to 4-month-old *HPRT*^+/−^ cloned rabbits. *HPRT*^+/−^ heterozygosity was confirmed by PCR, Southern blotting (Fig. 7A,B), and RT-PCR analyses (Supplementary Fig. S3). The DNA sequence of the RT-PCR product demonstrated that the mRNA was appropriately spliced at *HPRT* exon 2 and that exon 2 was linked to part of exon 3 and T2A-neo (Supplementary Fig. S4).

### Offspring from cloned HPRT^+/−^ rabbits

The five cloned *HPRT*^+/−^ rabbits were fertile when mated with wild-type male rabbits. A total of 38 kittens were born, with 17 offspring carrying the mutant *HPRT* gene, as revealed by PCR analysis (Supplementary Fig. S5). Ten female *HPRT*^+/−^ offspring survived. Seven male *HPRT*^*−*^offspring died before two months of age.

## Discussion

In addition to the mouse model, more animal models are needed to investigate other diverse human diseases. The rabbit is a traditional experimental animal used in various fields; therefore, the generation of gene knockouts or recombinant animals is desirable. Recently, genetic mutation in rabbits has been achieved by employing the ZFN^35^,^36^, TALENs^16^, and CRISPR/Cas9^18^,^37^ technologies. Here, we developed a gene-targeting approach that combines rAAV-mediated gene recombination and gene trapping to derive *HPRT* knockout fibroblasts. Different gene trap strategies were used in this study. The cloned rabbits were generated from the *HPRT* gene knockout fibroblasts by SCNT. This is the first report of gene targeting employing HR in the rabbit as a potential method for large gene fragment replacement.

In general, gene targeting by HR is inefficient in mammalian cells because of its dependence on DNA delivery techniques and its ability to undergo cell proliferation. It has been shown that rabbit fibroblasts can be stably cultured *in vitro* and used for SCNT after transgenic manipulation and antibody selection^24^. Delivery of the HR vectors to rabbit fibroblasts is the major barrier. We have introduced vectors with flanking 5-kb HR arms by electro- and liposome-mediated transfer, although no 6-TG-resistant cellular clones were identified (data not shown). Therefore, we selected the rAAV system to transfer the HR vectors. In the present study, male rabbit fibroblasts are susceptible to rAAV transformation. However, the induction rates were low when female fibroblasts were used for gene targeting. The differences in the transfection efficiencies due to the cell line status or gender are currently unclear.

When the T3SV vectors were delivered by rAAV, we obtained numerous G418-resistant clones, but none survived the 6-TG treatment. Consequently, a gene trapping strategy was introduced into the gene knockout system. Several types of gene trap strategies and technologies have been developed to enhance gene targeting^38^,^39^,^40^,^41^,^42^,^43^. We selected the promoter trap and polyA trap. IRES elements are generally used in promoter trapping or co-expression vectors. T2A is a new factor that has been used in co-expression experiments. When male fibroblasts were subjected to gene targeting, the G418-resistant clone frequencies were higher in the T3IR group, whereas the gene targeting frequencies were lower in the other groups (Table 1). It is possible that the IRES has a stop codon sequence that adapts to other open reading frames (ORFs) but that the T2A elements require a more accurate connection with other ORFs.

The *HPRT* locus is on the X chromosome of mammalian cells and has been used as a model gene to investigate genetic mutations in mammalian cell lines. The male *HPRT* knockout cells in the present study survived in a culture medium containing 6-TG. Of the 6-TG-resistant clones, most cell lines were positive for the mutation by PCR analysis using primers spanning the homologous arm. However, some were negative by PCR analysis, and the reasons underlying this finding are unclear. The Southern blotting results demonstrated that some male clones derived from the T3IR vector were complex, non-homogenous mixtures of wild-type and mutant cells (e.g., i4, i6, and i9) and that one clone (n1) from the T7NPA vector had a larger than expected band after restriction endonuclease digestion, although it is positive to probe 1 and probe 3. This suggests that such cells are not suitable for SCNT. We selected the mutant cells established using the T3T2A vector for use in SCNT. No kittens were born from 11 recipients, other than the four stillborn foetuses that were apparently retarded at 20 days of gestation. Subsequently, female rabbit fibroblasts were used for gene targeting.

The female G418-resistant clones were analysed by PCR because 6-TG could not be used for selection in *HPRT*^+/−^ cells. Fewer cell lines were *HPRT*^+/−^ (4 from the T3T2A vector, 3 from the T3IR vector). When the *HPRT*^+/−^ cell lines obtained using the T3T2A vector were used for SCNT, live kittens were derived, five of which developed to maturity. Tissues from the cloned rabbits were isolated for cell culture. Analysis by RT-PCR and Southern blotting demonstrated that all of the cloned rabbits were *HPRT*^+/−^. These results indicate that SCNT and rAAV-mediated gene knockout technologies can be combined to produce genetically manipulated rabbits.

In humans, the HPRT-deficient male shows the clinical disorder, which is characterized by dystonia, choreoathetosis, cognitive deficits, and self-injurious behaviour. However, there has been very little comprehensive characterization of the mechanisms underlying the *HPRT* mutation and the neurological abnormalities. Most clues about LND resulted from human HPRT-deficient cell lines^44^ or the *HPRT*^*−/−*^ mouse^45^, but the *HPRT*^*−/−*^ mice, even the *HPRT-APRT* deficient mice, did not exhibit any anatomical defects or spontaneous behavioural abnormalities^2^. The different behaviours of HPRT-deficient humans and mice may be related to the different purine metabolism pathways in the two species. In the present study, the male HPRT-deficient offspring from the *HPRT*^+/−^ rabbits did not survive for more than two months. This finding indicates that the male HPRT-deficient rabbits may potentially serve as an animal model for LND. Additional studies on the anatomical or behavioural abnormalities in the HPRT-deficient rabbits are thus warranted in the future.

X-chromosome inactivation is a universal phenomenon in mammals. The *HPRT* gene is located on the X-chromosome, one of which is randomly inactivated in female cells^46^. In eutherian mammals, such as rabbits and humans, the *Xist* homologue is not subject to imprinting, and X-chromosome inactivation begins later than that observed in mice. X-chromosome inactivation in human and rabbit embryos prior to implantation has been well studied; however, investigations involving post-implantation embryos are limited due to an insufficient number of markers^47^. However, in our investigations, the *Neo* gene was knocked into the *HPRT* gene and driven by the *HPRT* promoter, which should also serve as a good model for studying X-chromosome inactivation in embryos after implantation. In the present study, we only identified two cultured samples from the *HPRT*^+/−^ cloned rabbits that showed a high level of expression of the *HPRT-neo* mRNA (Supplementary Fig. S3). This phenomenon may result from different inactivation statuses in various parts of the body. During early embryo development, X-chromosomes are randomly inactivated, and X-chromosome inactivation was passed on to the daughter cells after mitosis. In the *HPRT*^+/−^ cloned rabbits, *HPRT-neo* was located on one X-chromosome. Thus, some skin tissues contained more cells derived from the *HPRT-neo* gene-activated cell, but others contained more cells derived from the *HPRT-neo* gene-inactivated cell. The level of *HPRT-neo* mRNA expression in the cultured cells depends on the percentage of *HPRT-neo* gene-activated cells in the biopsied tissues.

In conclusion, *HPRT* gene-targeted rabbits were generated by rAAV-mediated gene recombination and gene trapping through SCNT. The technique used in the present study may serve as a potential method for gene repair, conditional gene knockout, and gene knock-in to produce specific antibodies in rabbits.

## Methods

The Ethics Committee of the School of Medicine of Shanghai Jiao Tong University, approved all experimental protocols, including animal care, the SCNT protocol, embryo transfer, and Caesarean sections, which were in accordance with the approved guidelines (IACUC No.A-2013-001).

### Fibroblasts

Fibroblasts were isolated from 4- to 6-month-old adult male and female rabbits, as previously described^23^. The cells were cultured in FM (DMEM/F12 medium, Gibco, Grand Island, NY, USA) supplemented with 10% foetal bovine serum (FBS, Gibco) and cryopreserved at passage 3. The thawed fibroblasts were cultured and used for transfection within passage 8.

### rAAV HPRT targeting vector construction

The genomic DNA (gDNA) was isolated from male rabbit fibroblasts. The 5′ and 3′ HR arms were amplified from the rabbit genome by PCR (for primer sequences, see Supplementary Table S1) with a high fidelity polymerase (LA Taq Polymerase, TaKaRa, Dalian, China). The primers were designed based on the rabbit gDNA sequence (GenBank Acc. No. EF219063.1), and endonuclease sites for *Mlu*I, *Cla*I, *Bgl*II, and *Cpo*I were added to each primer. The 5′ and 3′ HR arms and the *Neo* transgene cassette were incised and ligated into the rAAV-hrGFP (Stratagene, Santa Clara, CA, USA) plasmid in the appropriate sequence. Four variants of neomycin phosphotransferase (*Neo*) cassettes derived from distinct plasmids were used as vectors: SV-neo consists of a *Neo* cDNA driven by the SV40 promoter, IRES-neo consists of a *Neo* cDNA adjacent to the IRES sequence, T2A-neo contains *Neo* cDNA connected to T2A, as previously reported^14^ (Fig. 1A), and NPA-neo comprises the *Neo* cDNA lacking a polyA tract (Fig. 1B). SV-neo, IRES-neo, and T2A-neo underwent replacement of the 3′ part of exon 3 and intron 3. Three plasmids were produced: T3SV, T3IR, and T3T2A. NPA-neo replaced the sequence comprising exons 7 and 8, and one plasmid was constructed and named T7NPA.

### Packaging rAAV-targeting constructs

Infectious rAAV stocks were produced with an AAV Helper-Free system (Stratagene), according to the manufacturer’s protocols. Briefly, the targeting constructs, pAAV-RC and pHELPER were expanded and the DNA was extracted using a QIAfilter Plasmid Midi kit (Qiagen GmbH, Hilden, Germany). Briefly, 10 μg of each of the three plasmid constructs (above) was mixed and transferred into 70% confluent 293A cells at passages 7–10 in a 75 cm^2^ flask using the calcium phosphate cell transfection method. Three days after transfection, the cells were collected into 1 mL of DMEM/F12, frozen in liquid nitrogen, and then thawed in a 37 °C water bath; the process was repeated three times. The crude lysate was clarified by centrifugation, and the supernatant passed through a 0.22-μm filter. The viral stocks were stored at −80 °C before use.

### Packaged rAAV infection and selection

Adult fibroblasts were cultured in FM and used for infection when the cells were 75% confluent. Approximately 2 × 10^5^ cells were trypsinized and collected into 15 mL tubes. The supernatants were removed after centrifugation, and the cell pellets were resuspended in a 0.25-mL viral stock and then incubated at 37 °C for 10 min. Approximately 2 mL of DMEM/F12 supplemented with 10% knockout SR (Gibco) was added to the tube, and the cell suspension was transferred into a 35-mm culture dish. The culture media were replaced with FM 8 h after viral treatment. After 24 h, the cells were trypsinized and transferred into four 48-well plates. After 2 days, G418 (1,000 μg/mL, Sigma) was added to the culture medium. Ten days later, the cell clones were trypsinized and transferred into a new well for cell propagation. When each cell line was transferred into four wells of a 24-well plate, 6-TG (50 μg/mL, Sigma) was added to the culture medium of one well of the male fibroblasts. For each cell line, a portion of the cells were cryopreserved for future use, and some were further propagated for PCR screening and Southern and western blotting analyses.

### PCR and RT-PCR analyses

The gDNA was isolated from cells grown to confluence in 35-mm dishes using a TIANamp Genomic DNA Extraction Kit (Tiangen, China). The sequences encompassing the HR arm were amplified using the primers listed in Table S1. The PCR products were electrophoresed on 1.0% agarose gels. The RNA was extracted from confluent cells using a Transcriptor First Strand cDNA Synthesis kit (Roche, Mannheim, Germany). The primers were designed against the mature, spliced mRNA. The target sequence spans *HPRT* exon 2 to the *Neo* cassette. A portion of the RT-PCR products was sequenced by Invitrogen.

### Southern blotting

The phenol-chloroform-isopentanol method was used to extract the DNA from the PCR-positive fibroblast cell lines and the cloned rabbits. The *Neo* DNA probe (Probe 1) and the probes directed against the sequence outside the HR arms (Probes 2 and 3) were amplified from the pcDNA3 plasmid and the gDNA using the primers listed in Table S1. The amplified DNA fragments (300 ng) were labelled using a random-primed labelling technique (DIG High-Prime DNA Labeling and Detection Starter Kit I (Roche, Germany). The gDNA (20 μg) from the T3IR- and T3T2A-targeted clones was digested using *EcoT22*I (TaKaRa) and *Hind*III, respectively. The gDNA from T7NPA-targeted clones was digested using *Hpa*I for probe 1, and *EcoR*V and *EcoR*I for probe 3. A 0.8% agarose gel was loaded with the gDNA samples and electrophoresed at 4 V/cm for 4 h. Membrane transfer was conducted using an iBlot dry blotting system (Invitrogen, Israel). After pre-hybridization, the probe was added and hybridized at 54 °C–56 °C. Twenty-four hours later, the membranes were washed and blocked, and the hybridized probe was immunodetected using anti-digoxigenin-AP and NBT/BCIP, according to the manufacturer’s protocols.

### Western blotting

The targeted cells (~1 × 10^7^) were resuspended in 200 μL of a radioimmunoprecipitation assay (RIPA) lysis buffer (Biyuntian, China) with PMSF, placed on ice for 30 min, according to the protocol, and then centrifuged at 14,000 *g* for 20 min at 4 °C. The cell lysates were subjected to SDS–PAGE, transferred to PVDF membranes (Millipore, Darmstadt, Germany), and incubated with an anti-HPRT antibody (ab10479, Abcam, United Kingdom). A horseradish peroxidase-conjugated secondary antibody was applied for 1 h. After extensive washing, the protein bands were visualized with the Novex® ECL Chemiluminescent Substrate Reagent Kit (Invitrogen, USA) using an ImageQuant LAS4000 mini (GE Life Sciences). The membranes were re-probed with antibody to glyceraldehyde-3-phosphate dehydrogenase (GAPDH) as a loading control.

### SCNT and embryo transfer

The SCNT procedure was conducted as previously described^23^, with some modifications. Briefly, the oocytes were enucleated by removing the first polar body and the associated metaphase plate under an inverted microscope equipped with a spindle view system (Cambridge Research & Instrumentation Inc., USA). Serum-starved *HPRT* knockout fibroblasts cultured in DMEM/F12 medium supplemented with 0.5% FBS for 2–4 days were used as donor cells. The reconstructed embryos were electrofused in a fusion buffer using two pulses (25 μs DC, 2.0 KV/cm). The SCNT embryos were activated after 30 min by a second identical set of electric pulses and then cultured in mRD supplemented with 5 μg/mL cycloheximide and 2 mM 6-dimethylaminopurine for 1 h. The embryos were then incubated with mRD for 1 h, followed by incubation in B2 medium that was prepared as previously described^48^ and supplemented with 5% FBS. The reconstructed embryos were transferred to pseudopregnant recipients. Pregnancies were assessed after 14 and 29 days by palpation. Caesarean section was performed 31 days after embryo transfer.

### Analysis of the HPRT^+/−^ rabbits

Fibroblasts were isolated from 3- to 4-month-old *HPRT*^+/−^ rabbits, as previously described^23^. The fibroblasts were cultured for subsequent Southern blotting of the gDNA and RT-PCR analyses. At maturity, the cloned female *HPRT*^+/−^ rabbits were mated with *HPRT* wild-type male rabbits.

## Additional Information

**How to cite this article**: Yin, M. *et al.* Generation of hypoxanthine phosphoribosyltransferase gene knockout rabbits by homologous recombination and gene trapping through somatic cell nuclear transfer. *Sci. Rep.* **5**, 16023; doi: 10.1038/srep16023 (2015).

## Supplementary Material

Supplementary Information

## Figures and Tables

**Table 1:** rAAV-mediated gene knockout in rabbit fibroblasts.

Cell line	Vector	Cells	G418-resistant clones (%)	6-TG-resistant clones	Targeted/G418-resistant clones (%)
Male	T3SV	4 × 10^5^	91(0.02)	2	0(0)
	T3T2A	4 × 10^5^	107(0.03)	28	25(23.36)a
	T3IR	5 × 10^4^	73(0.14)	9	7(9.59)b
	T7NPA	4 × 10^5^	84(0.02)	5	4(4.76)b
Female	T3T2A	2 × 10^5^	10(0.005)	/	4(40)
	T3IR	6 × 10^5^	14(0.0023)	/	3(21.4)
	T7NPA	2 × 10^5^	17(0.0085)	/	0

Chi-squared analyses demonstrate significant differences between a and b (P > 0.05).

**Table 2:** Production of HPRT gene knockout rabbits by SCNT using different cell lines.

Cell line	Embryos transferred	Recipients	Full term rabbits	Alive kits at birth	Alive rabbits at weaning
MT3T2A	276	11	0*		
FT3T2A	784	38	24	18	5

^*^Four stillborn foetuses were retarded at gestation day 20.
