# Supplementary material for: Generation of hypoxanthine phosphoribosyltransferase gene knockout rabbits by homologous recombination and gene trapping through somatic cell nuclear transfer
Source: Sci Rep. 2015 Nov 2;5:16023. doi: 10.1038/srep16023 (PMC4629196; doi:10.1038/srep16023)
Supplement: Supplementary Information [file srep16023-s1.pdf]

Title

Generation of hypoxanthine phosphoribosyltransferase gene knockout rabbits by homologous recombination and gene trapping through somatic cell nuclear transfer

Mingru Yin[#], Weihua Jiang[#], Zhenfu Fang, Pengcheng Kong, Fengying Xing, Yao Li, , Xuejin Chen* and Shangang Li*

Supplementary information

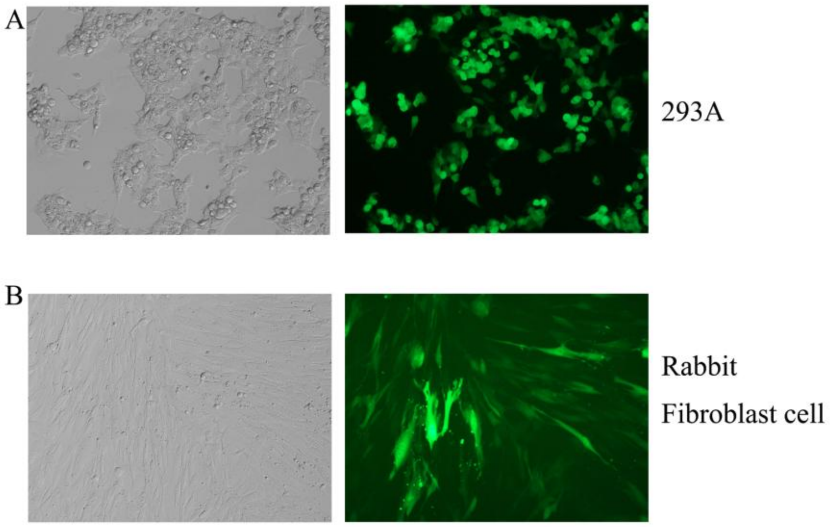

Fig. S1 Packaged and Infection of rAAV-hrGFP vector

(A) The prAAV-hrGFP vector was packaged in 293A cells; left, bright field; right, GFP fluorescence.

(B) The packaged pAAV-hrGFP virus infected rabbit fibroblasts; left, bright field; right, GFP fluorescence.

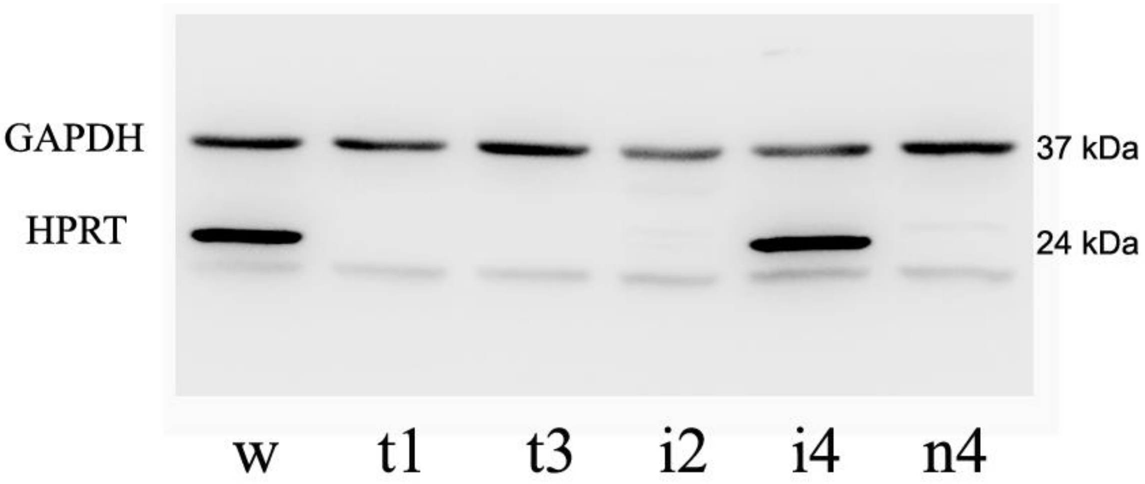

Fig. S2 Western blot analysis of *HPRT* knock-out cell lines.

Cell line t1, t3 (T3T2A); i2, i4 (T3IR); n4 (T7NPA). W, protein of wild-type rabbit fibroblasts. GAPDH was used as a gel loading reference. HPRT band size is about 24 kDa, GAPDH band size is about 37 kDa.

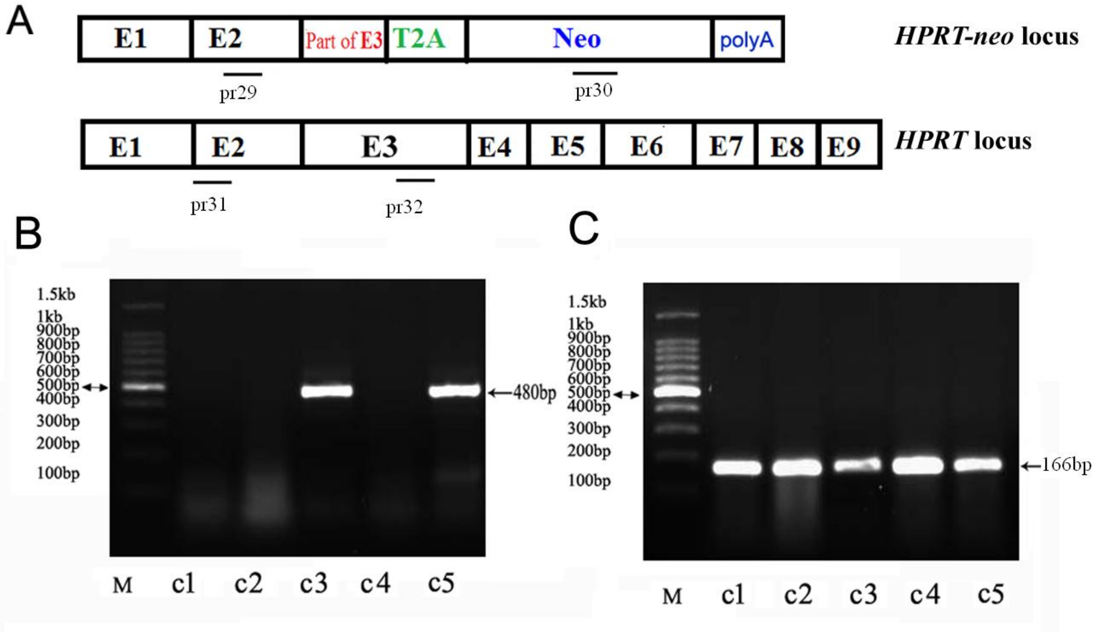

Fig. S3 RT-PCR analysis of five cloned *HPRT*$^{+/-}$ rabbits.

(A) The locus of primers (listed in TableS1) used for RT-PCR.

(B) RT-PCR analysis of *HPRT-neo* locus; arrow indicates the target fragments approximately 480 bp.

(C) RT-PCR analysis of *HPRT* locus; indicated by band of approximately 166bp.

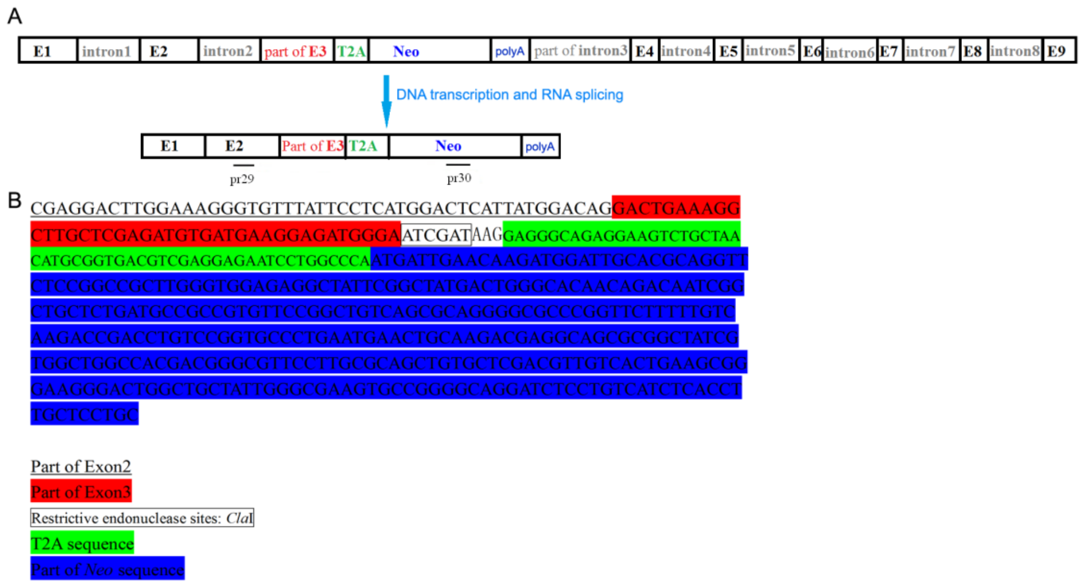

Fig. S4 DNA sequence analysis of the *HPRT-neo* locus

(A) Schematic diagram of the *HPRT-neo* locus and RNA splicing.

(B) DNA sequence of RT-PCR product.

TableS1. Primers used in the research

Target	Primer name	Primer sequence	No.
EX35'-Homo-arm from genome	EX3LFMluI	CATT*ACGCGT*TTGTCCTGTAGTTCTAACATTCA	Pr1
	EX3LFClaI-IRES with stop code	TAAC*ATCGAT*TCAGTCATTTATCATCCCATCTCCTTCATCACATCT (REV)AGATGTGATGAAGGAGATGGGATGATAAATGACTGA*ATCGAT*GTTA	Pr2
	EX3LRClaI	TAAC*ATCGAT*TCCCATCTCCTTCATCACATCT	Pr3
EX33'-Homo-arm from genome	EX3RFBglII	TCAA*AGATCT*TAGTTTTCCTGTTTTCATTCTT	Pr4
	EX3RRCpoI	CGAA*CGGTCCG*ACCGAAATAAAATGTCAGTGTT	Pr5
EX75'-Homo-arm from genome	EX7LFMluI	CATT*ACGCGT*CTCTTCCACCAATCCCAACTCT	Pr6
	EX7LRClaI	TAAC*ATCGAT*ACTACAGGGCATTACCAAAGAG	Pr7
EX73'-Homo-arm from genome	EX7RFBglII	TCTT*AGATCT*TTCTTCTTCTTCTCGCTCTTAT	Pr8
	EX7RRCpoI	CGAA*CGGTCCG*TCCACCCAAAGGGAACTGATAG	Pr9
IRES pIGCN21	IRESFClaI	AAG*ATCGAT*AAGCTATCCAATTCCGCCCCCCCC	Pr10
	IRESRBglII	TCAA*AGATCT*ACGTTAAGGGATTTTGGTCATG	Pr11
SVneo From PL452	SVneoFClaI	GAAC*ATCGAT*AGCCCAATTCCGATCATA	Pr12
	SVneoRBglII	CCGT*AGATCT*TGTAAACGACGGCCAGT	Pr12
T2Aneo from PCDNA3	T2AmegFClaI	GGGA*ATCGAT*AAGGAGGGCAGAGGAAGTCTGCTAACATGCGGTGAC	Pr14
	T2AmegF	AACATGCGGTGACGTCGAGGAGAATCCTGGCCCA*ATGATTGAACAA GATGGATTGCACG*	Pr15
	IRESRBglII	TCAA*AGATCT*ACGTTAAGGGATTTTGGTCATG	Pr16
NPA-neo from PCDNA3	NP-neoFClaI	GAAC*ATCGAT*CTGTGGAATGTGTGTCA	Pr17
	NP-neoRBglII	CCGT*AGATCT*GCGGTGGAATCGAAATCT	Pr18
Knockout analysis for EX3	InneoF	CTCTGATGCCGCCGTGTT	Pr19
	OutE33'arm	TGTCTCCCTCCCTGACTTTTG	Pr20
Knockout analysis for EX7	OutE75'arm	TTACCTCAGAGCCAGTTGTCG	Pr21
	InneoR	GAGTAGTCCTCCTCATCTTCCTTG	Pr22
Probe for neo	Neo probeF	GTCACTGAAGCGGGAAGGG	Pr23
	Neo probeR	CGGCGATACCGTAAAGCAC	Pr24
Probe out of EX3 homo-arm	OutEX3probeF	CTCTTTAATGTGGCTTGA	Pr25
	OutEX3probeR	AATAAACACCCTTTCCAA	Pr26
Probe out of EX7 homo-arm	OutEX7probeF	GAAGGAAGCCCTCTAACC	Pr27
	OutEX7probeR	GTAACAGCCAACAATGAAAA	Pr28
mRNA of *hprt-neo*	RT *hprt-neo*F	CGAGGACTTGGAAAGGGTGT	Pr29
	RT *hprt-neo*R	GCAGGAGCAAGGTGAGATGAC	Pr30
mRNA of *hprt*	Q*hprt*F	AGTGATGATGAACCGGGATA	Pr31
	Q*hprt*R	AGAGGGCTACAATGTGATGG	Pr32
SRY	*SRY*F	GCACAGCGTGGAAGTAGGT	Pr33
	*SRY*R	TGCGAAACTCAGACATCAGC	Pr34

Red: Restrictive endonuclease sites

Blue: Stop codons
